# Effect of propolis and N-acetylcysteine supplementation on lipoprotein subclasses distribution and paraoxonase 1 activity in subjects with acute respiratory infection

**DOI:** 10.5937/jomb0-24695

**Published:** 2020-10-02

**Authors:** Jelena Vekić, Jasmina Ivanišević, Aleksandra Zeljković, Vesna Spasojević-Kalimanovska, Nataša Bogavac-Stanojević, Marija Mihajlović, Jelena Janać, Sanja Vujčić, Milica Miljković, Dejan Zujović, Jelena Kotur-Stevuljević

**Affiliations:** 1 University of Belgrade, Faculty of Pharmacy, Department of Medical Biochemistry, Belgrade; 2 Municipal Institute for Lung Disease and Tuberculosis, Belgrade

**Keywords:** acute respiratory infection, antioxidants, lipoproteins, N-acetylcysteine, propolis, smoking, pušenje, propolis, N-acetilcistein, lipoproteini, antioksidanti, akutna respiratorna infekcija

## Abstract

**Background:**

Propolis and N-acetylcysteine have positive impact on respiratory tract health. Also, it has been suggested that they have beneficial effects on serum lipid and oxidative stress status, but the available data are limited and mostly gained from animal models. In this study we evaluated the effects of propolis and N-acetylcysteine supplementation (PropoMucil®) on lipid status, lipoprotein subclasses distribution and paraoxonase 1 activity in subjects with acute respiratory infection.

**Methods:**

Twenty subjects with acute respiratory infection were included. PropoMucil® granules for oral solution (80 mg of dry propolis extract and 200 mg of N-acetylcysteine) were administered tree times per day for ten days. Serum lipid profile, paraoxonase 1 activity and low-density and high-density lipoprotein size and subclasses distribution were assessed at baseline and after supplementation.

**Results:**

Following ten days of supplementation lipid status remained unchanged, but a significant increase of low-density lipoprotein particle size and proportion of high-density lipoprotein 3a particles were found (P<0.05). Moreover, supplementation with PropoMucil® significantly improved high-density lipoprotein particles distribution, particularly in those who smoke. There was a moderate increase of paraoxonase 1 activity, but without statistical significance.

**Conclusions:**

The presented study demonstrated that short-term supplementation with PropoMucil® has beneficial effects on low-density and high-density lipoprotein subclasses distribution and paraoxonase 1 activity in subjects with acute respiratory infection particularly in those who smoke.

## Abstract (sr)

**Uvod:**

Propolis i N-acetilcistein pozitivno utiču na zdravlje disajnih puteva. Takođe, sugeriše se da oni imaju blagotvorno dejstvo na lipidni i oksidativno-stresni status, ali podaci su ograničeni i dobijeni uglavnom na životinjskim modelima. U ovom istraživanju, procenili smo kombinovani efekat propolisa i N-acetilcisteina (PropoMucil®) na status lipida, raspodelu supklasa lipoproteina i aktivnost paraoksonaze 1 kod pacijenata sa akutnom respiratornom infekcijom (ARI).

**Metode:**

Uključeno je 20 ispitanika sa akutnom respiratornom infekcijom. Oralni rastvor praška PropoMucil® (80 mg suvog ekstrakta propolisa i 200 mg N-acetilcisteina) je aplikovan 3 puta dnevno tokom 10 dana. Serumski lipidni profil, aktivnost praoksonaze 1 i veličina i raspodela supfrakcija lipoproteina niske gustine i lipoproteina visoke gustine su određeni pre i nakon suplementacije.

**Rezultati:**

Nakon deset dana suplementacije, lipidni status je ostao nepromenjen, ali je utvrđeno značajno povećanje veličine lipoproteina niske gustine i relativnog udela lipoproteina visoke gustine 3a (P <0,05). Pored toga, suplementacija PropoMucil®-om značajno je poboljšala distribuciju lipoproteinskih čestica visoke gustine, posebno kod pacijenata koji puše. Došlo je do umerenog porasta aktivnosti paraoksonaze 1, ali bez statističke značajnosti.

**Zaključak:**

Studija je pokazala da kratkotrajna suplementacija PropoMucil®-om ima korisne efekte na distribuciju supklasa lipoproteina niske i visoke gustine i aktivnost paraoksonaze 1 kod ispitanika sa akutnom respiratornom infekcijom, posebno kod onih koji puše.

## Introduction

Acute respiratory infections (ARI) are the most common infective diseases of respiratory tract and most frequently have viral aetiology [1]. The detrimental effects of tobacco smoke on respiratory tract health and predisposition of smokers for development of ARI are also well recognised [2].

Acute infection and inflammation affect lipoprotein metabolism, structure and functionality [3]. In particular, serum lipid profile in acute phase response is characterised by elevated triglycerides (TG) and reduced high-density lipoprotein cholesterol (HDL-C) levels, while the concentration of total (TC) and lowdensity lipoprotein cholesterol (LDL-C) might be normal or slightly elevated. In line with previous, similar observations are reported in ARI [4]. As chronic, lowgrade inflammation leads to profound disorders of lipoprotein metabolism and dyslipidemia [5] and ARI and smoking may be associated with chronic pulmonary disease or chronic inflammatory state, disturbances in lipoprotein metabolism are more expected to occur. Lipid peroxidation of LDL particles is the common link between oxidative stress and dyslipidemia and the initial step in atherogenesis [6]. Another important link is paraoxonase 1 (PON1), an enzyme associated with HDL particles, which protects LDL against lipid peroxidation [7].

In recent years, a great attention has been focused on the use of functional food and dietary supplements with antioxidant and hypolipidemic activity [8] [9]. Propolis is a natural product and complex mixture of various bioactive compounds, which is traditionally used to prevent or assist recovery from the infection [10]. Although based on limited data, propolis supplementation showed beneficial effects in the management of chronic diseases, such as diabetes [11] and certain types of cancers [12]. A recent double-blind, placebo-controlled study showed significant improvement of oxidative stress and lipid status following 90 days of propolis supplementation [13]. Yet, the effects of propolis on lipoprotein size and subclasses profile and PON1 activity are still unknown. One of most effective application of N-acetylcysteine (NAC) is the therapy of respiratory diseases, due to its mucolytic properties [14]. In addition, NAC stimulates biosynthesis of glutathione (GSH) and has a direct antioxidative role as free radical scavenger [14]. It seems that NAC supplementation might have beneficial effects on serum lipid and PON1 status, but the available data is limited and mainly obtained from animal models and in vitro studies [15] [16].

Both, propolis and NAC have positive impact on respiratory tract health and the efficiency of their specific combination, PropoMucil® (Abela Pharm, Belgrade, Serbia), was recently reported in children and adolescents with ARI [17]. However, no previous study investigated the effects of this supplement on lipid status. In the current study we conducted a short-term supplementation (as indicated for acute disease process) of 20 subjects with ARI with PropoMucil®. The supplementation period corresponds to the data of the previous study, demonstrating a significant improvement of ARI symptoms with the same combination of propolis and NAC in majority of pediatric patients after 10 days [17]. The aim of this study was to assess the effects of propolis and NAC (PropoMucil®) supplementation on lipid status, lipoprotein size and subclasses distribution and PON1 activity in subjects with ARI.

## Materials and Methods

### Study subjects and protocol

The study included 20 subjects, 7 men and 13 women, aged 62.5 ± 15.0 years, who were referred to the Municipal Institute for Lung Disease and Tuberculosis, Belgrade, Serbia and, after clinical and laboratory examination, diagnosed with ARI (acute bronchitis). Among them, 30% were smokers. For all the subjects, microbiological analysis of sputum and C-reactive protein (CRP) concentration testing were performed. The results of sputum analysis were negative for bacteria and CRP concentrations indicated a viral infection.

PropoMucil® 200 powder for oral solution (Abela Pharm, Belgrade, Serbia), containing 80 mg of dry propolis extract and 200 mg of NAC, were administered tree times per day for 10 days to each subject. According to the manufacturer specification, PropoMucil® contains purified dry propolis extract (20%), standardized to 12% of total polyphenols, with 20% of NAC. Propolis extract was obtained by dynamic multi extraction technology (MED) and does not contain resins, but is enriched in integral polyphenols (phenolic acids, flavonoids, aglycons and glucosides) [18].

All study participants completed 10 days supplementation period and reported no allergy or any other adverse effects. None of the subjects included in the study received any other medications for ARI, lipid-lowering therapy or antioxidant supplementation at baseline and during the follow-up. At baseline and following the supplementation the data on the presence/absence of cough and expectoration, as well as sputum characteristics (clear/purulent) were recorded. Blood samples were also collected at baseline and after 10 days of supplementation. At each time point the blood was drawn after a 12-hour fasting period into one evacuated tube containing EDTA and one serum sample tube. The study was planned according to the ethical guidelines following the Helsinki Declaration and approved by the Ethics Committee at the Faculty of Pharmacy, University of Belgrade and Municipal Institute for Lung Disease and Tuberculosis. Informed consent was obtained from all study participants.

### Laboratory analyses

Serum lipid parameters (TC, LDL-C, HDL-C and TG) were measured by routine laboratory methods and the concentration of CRP was measured by immunoturbidimetric assay (Biosystems S.A., Bar celona, Spain) using an ILab300+ analyser (Instrumentation Laboratory, Milan, Italy). Plasma LDL and HDL subclasses were separated by gradient gel electrophoresis method, as it has been published elsewhere [19]. This method provides simultaneous determination of LDL and HDL particle sizes and relative proportions of seven LDL and five HDL subclasses. The proportion of small, dense LDL particles (sdLDL) was estimated by summing up relative proportions of LDL III and IV subclasses [19]. The activity of PON1 was measured as a rate of paraoxon hydrolysis, using the method developed by Richer and Furlong [20], as modified in our laboratory and published elsewhere [7].

### Statistical analysis

Data are shown as mean ± standard deviation for normally distributed variables and as median and lower and upper limits of interquartile range (IQR) for non-normally distributed variables. Categorical variables are presented as absolute and relative frequencies and compared by Chi-square test. We tested the normality of all continuous variables, using Shapiro-Wilk test. Non-normally distributed data were further log transformed, but they failed to achieve normality, and therefore were analysed by non-parametric tests. Comparisons of baseline and the data after 10 days of supplementation were performed by the parametric paired t-test for normally distributed data or by the paired Wilcoxon's signed-rank test for non-normally distributed variables. The unpaired Mann-Whitney Exact test was used to compare relative changes in laboratory data between smokers and non-smokers. Correlation analysis was performed by Spearman's rank correlation method.

## Results

After 10 days of supplementation with PropoMucil® all subjects reported improvement of the symptoms, reflected by reduced intensity of cough in 17 subjects (85%) and reduced expectoration in 14 patients (70%). The number of patients having cough was significantly reduced (100% at baseline vs. 70% after supplementation; P<0.01), as well as the number of patients with purulent sputum (30% at baseline vs. 0% after supplementation; P<0.01).

Table 1 shows serum lipid parameters and PON1 activity at baseline and after 10 days of supplementation with PropoMucil® in all ARI patients. All evaluated parameters of lipid status remained unchanged after supplementation. Also, we found a moderate increase in PON1 activity after supplementation which was not statistically significant. Finally, there was a modest decrease in CRP levels after supplementation, but the difference did not reach statistical significance.

**Table 1:** Effect of supplementation on serum lipid parameters, PON1 activity and CRP concentration in all ARI patients. Data were compared by parametric paired t test and presented as mean ± standard deviation. # Data were compared by paired Wilcoxon’s signed-rank test and presented as median (lower and upper limit of interquartilerange).

	Baseline	After 10 days	P
TC, mmol/L	4.89 ± 0.51	4.96 ± 0.39	0.339
LDL-C, mmol/L	3.16 ± 0.49	3.19 ± 0.44	0.598
HDL-C, mmol/L	1.45 ± 0.31	1.49 ± 0.30	0.605
TG, mmol/L#	0.56 (0.49–0.71)	0.60 (0.48–0.79)	0.465
PON1, U/L#	247.0 (226.5– 472.0)	256.0 224.5–507.0)	0.763
CRP, mg/L#	2.10 (1.20–3.10)	2.0 (1.05–2.85)	0.919

Next, we evaluated the effects of supplementation on LDL and HDL particles characteristics (Table 2) . After 10 days of PropoMucil® supplementation a significant increase of LDL particles size was found. In addition, the proportion of small HDL 3a particles was significantly higher following supplementation period.

**Table 2:** Effect of supplementation on LDL and HDL size and subclasses in all ARI patients. Data were compared by parametric paired t-test and presented as mean ± standard deviation.

	Baseline	After 10 days	P
LDL size, nm	27.43 ± 0.63	27.64 ± 0.63	<0.05
LDL I, %	24.8 ± 3.8	23.7 ± 2.7	0.255
LDL IIA, %	11.3 ±1.5	11.0 ± 1.2	0.546
LDL IIB, %	14.0 ± 1.1	13.9 ± 1.7	0.758
LDL IIIA, %	12.7 ± 1.1	12.9 ± 1.0	0.564
LDL IIIB, %	7.2 ± 1.3	7.1 ± 0.9	0.799
LDL IVA, %	12.2 ± 2.3	13.0 ± 2.1	0.214
LDL IVB, %	18.2 ± 3.1	18.3 ± 2.6	0.785
sdLDL, %	50.2 ± 5.2	51.3 ± 4.1	0.320
HDL size, nm	9.82 ± 1.05	10.06 ± 0.96	0.176
HDL 2b, %	39.9 ± 4.81	38.88 ± 6.86	0.232
HDL 2a, %	22.1 ± 3.1	22.3 ± 1.8	0.688
HDL 3a, %	16.3 ± 2.1	17.3 ± 0.7	<0.05
HDL 3b, %	11.2 ± 0.5	11.2 ± 0.6	0.965
HDL 3c, %	10.5 ± 0.6	10.3 ± 0.8	0.664

In further analysis, we evaluated possible associations between relative changes of biochemical parameters and lipoprotein size and subclasses distribution. Spearman's correlation analysis showed that relative changes in TG levels after supplementation were significantly inversely associated with the changes in HDL 2b subclasses proportion (r = -0.499; P=0.025), but positively with changes in the proportion of HDL 3a particles (r = 0.460; P=0.041). The changes in CRP levels significantly positively correlated with the changes in proportions of small LDL III particles (r = 0.638; P=0.038).

**Figure 1:**
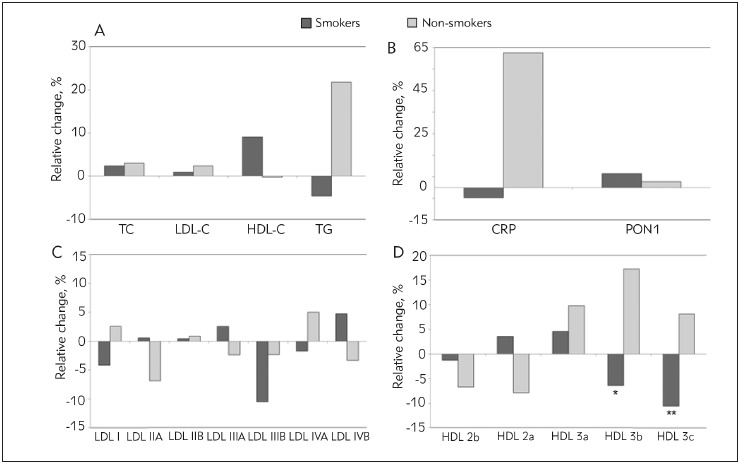
Relative changes of laboratory parameters after 10 days of supplementation in ARI smokers and non-smokers. A, Serum lipid parameters; B, CRP concentration and PON1 activity; C, LDL subclasses; D, HDL subclasses. Data are compared by the Mann-Whitney Exact test and presented as medians: * P<0.05; ** P<0.01.

The effects of 10 days of supplementation on serum lipid profile (Figure 1A) were more prominent in the smokers, as indicated by increase of HDL-C (median relative change of +9% in smokers vs. -0.3% in non-smokers) and reduction of TG (median relative change of -4.6% in smokers vs. +21.8% in non-smokers), although with no significance. In addition, the smokers with ARI experienced beneficial effects on CRP and PON1 levels (Figure 1B). The supplementation showed opposite effects on HDL subclasses distributions in smokers and non-smokers. In particular, the proportions of small HDL 3b and HDL 3c particles were significantly reduced after supplementation in smokers with ARI (Figure 1D). Similarly, the changes in LDL subclasses distribution was more pronounced in smokers with ARI, yet without significance (Figure 1C).

## Discussion

The results of the presented study demonstrated that PropoMucil® has some beneficial effects on LDL and HDL subclasses distribution in subjects with ARI. Following 10 days of supplementation general lipid status remained unchanged, but more depth analysis showed that HDL 3 particles distribution was significantly improved, particularly in those who smoke. The supplementation with PropoMucil® did not significantly affect PON1 activity.

Propolis is a mixture of various bioactive compounds that contribute synergistically to its overall effects [10]. Regarding serum lipid profile, data from animal models showed that propolis reduced LDL-C, while increased HDL-C levels in the rabbits fed a cholesterol-enriched diet [21]. Available data suggest that NAC also possess certain hypolipidemic effects [15], probably as a consequence of improved insulin sensitivity following NAC administration [22].

Although serum lipid parameters did not significantly change after 10 days of supplementation (Table 1), beneficial effects on serum lipoprotein profile was shown (Table 2 and Figure 1). There was a significant increase in LDL size after 10 days of supplementation (Table 2), while the effect on LDL subclasses distribution was more pronounced in the smokers with ARI (Figure 1). It has been reported that quercetin, one of the most common flavonoid in propolis, may upregulate LDL receptors synthesis in HepG2 cells via protein kinases cascade activation of sterol regulatory element binding protein-2 (SREBP-2) [23]. Increased receptors expression could be one of the reasons for faster sequestration of pro-atherogenic sdLDL particles from the circulation thereby increasing the proportion of larger LDL particles and dominant LDL size.

Plasma HDL populations are constituted by several subfractions that differ in size, density and composition, ranging from HDL 3c, as the smallest and the most dense, to HDL 2b, which are the largest and the least dense particles. More importantly, different HDL subclasses have distinct metabolic behaviour and anti-atherogenic properties [24]. Data of our and other groups consistently showed decreased proportion of large HDL 2 and/or the abundance of smaller HDL 3 particles in different categories of patients with increased cardiovascular risk [25]. Namely, smaller HDL 3 particles, although essentially considered as atheroprotective, might undergo structural changes in pro-inflammatory conditions, which further compromise their functionality, as it was documented in respiratory disease, too [26] [27]. In line with previous, our data showed that supplementation with PropoMucil® significantly improved HDL particles distribution, as reflected by significant reduction of smaller HDL 3 subclasses proportion in those who smoke (Figure 1). Our results might be explained by flavonoid-mediated regulation of some pathways in lipoprotein metabolism. It was demonstrated that rutin, another abundant flavonoid in propolis, increases the activity of lecithin: cholesterol acyltransferase (LCAT), an enzyme which mediates maturation of HDL particles in plasma [28] leading to subsequent reduction of smaller HDL particles. Also, changes in the proportion of small HDL 3 particles in our study were related to the changes in TG levels following supplementation. All of the above may account for more pronounced effects of the supplementation on HDL subclasses distribution in smokers with ARI.

PON1 is a major antioxidant component of HDL particles with an ability to hydrolyze lipid peroxides, thus protecting LDL particles from oxidation [7]. ARI [3] and tobacco smoking [29] can contribute to diminished enzyme activity. However, the inhibition is reversible and PON1 activity can be restored by antioxidants, such as NAC, or by caffeic acid phenetyl ester (CAPE), a component of propolis [29] [30]. Our results showed no significant difference in PON1 activity at baseline and following supplementation with PropoMucil® (Table 1). In the recent study by Shen et al. [16], NAC administration in the group of rats exposed to polychlorinated biphenyls (PCB) caused a significant increase in serum PON1 activity, whereas such effect was not found in the control group, suggesting potential interaction of PCB and NAC on PON1 activity. Also, although there is evidence in both cell cultures and animal models that quercetin upregulates PON1 gene expression [31], quercetin supplementation in humans had no significant effects on PON1 activity [32]. The observed trend of greater increase of PON1 activity in smokers indicate that additional studies, with more subjects included, are necessary to elucidate the potential effects of complex mixture of NAC and propolis supplementation on PON1 activity.

A potential limitation of our study could be a lack of a control or placebo group and small number of subjects included. Nevertheless, a current study is one of the few investigations of joined propolis and NAC effects on lipid metabolism in humans and all laboratory analyses were performed blindly, which accounts for its strength. A relatively short supplementation period could be a reason for the lack of difference in serum lipid parameters before and after supplementation. However, the observed subtle changes in lipoprotein particles distribution indicate that this supplementation period was sufficient to ascertain the effectiveness of the supplement. Additional studies, designed for dyslipidemic patients, are encouraged to verify our preliminary observations.

In conclusion, the current study provides the first data on positive effects of PropoMucil® supplementation on plasma lipoprotein size and subclasses distribution and PON1 activity in subjects with ARI with more evident effects of supplementation obtained in the group of smokers.

## Author contributions

JV: designed the work, interpret the results, drafted the manuscript, approved the final version, agreed to be accountable for all the aspects of the work; JI: acquired data, interpret the results, drafted the manuscript, approved the final version, agreed to be accountable for all the aspects of the work; AZ: designed the work, interpret the results, revised the manuscript, approved the final version, agreed to be accountable for all the aspects of the work; VSK: designed the work, revised the manuscript, approved the final version, agreed to be accountable for all the aspects of the work; NBS: designed the work, interpret the results, revised the manuscript, approved the final version, agreed to be accountable for all the aspects of the work; MM: acquired data, revised the manuscript, approved the final version, agreed to be accountable for all the aspects of the work; JJ: acquired data, revised the manuscript, approved the final version, agreed to be accountable for all the aspects of the work; SV: acquired data, revised the manuscript, approved the final version, agreed to be accountable for all the aspects of the work; MM: acquired data, revised the manuscript, approved the final version, agreed to be accountable for all the aspects of the work; DZ: designed the work, acquired data, revised the manuscript, approved the final version, agreed to be accountable for all the aspects of the work; JKS: designed the work, interpret the results, revised the manuscript, approved the final version, agreed to be accountable for all the aspects of the work.

*Acknowledgements*. This investigation wasfinancially supported by the Innovation Fund Republic of Serbia, Innovation vouchers Programme No.152/2018 and by the Ministry of Education, Science and Technological Development of Serbia, Project No. 175035.

## Conflict of interest statement

The authors declare that there is no conflict of interest. Abela Pharm provided the supplement, but did not have any impact on its testing and the results.

## List of abbreviations

ARI, acute respiratory infection; CAPE, caffeic acid phenetyl ester; CRP, C-reactive protein; EDTA, ethylen diamino tetraacetic acid; GSH, glutathione; HDL, high density lipoprotein; IQR, interquartile range; LCAT, lecithin: cholesterol acyltransferase; LDL, low density lipoprotein; MED, multi extraction technology; NAC, N-acetylcysteine; PCB, polychlorinated biphenyls; PON1, paraoxonase 1; sdLDL, small dense LDL; SREBP2, sterol regulatory element binding protein2; TC, total cholesterol; TG, triglycerides.
